# Transition of Care From Pediatric to Adult Services for Patients With Anorectal Malformations: A Qualitative Study

**DOI:** 10.1002/wjs.70467

**Published:** 2026-06-25

**Authors:** Leila Hartford, Niveshni Maistry, Giulia Brisighelli, Juan Scribante

**Affiliations:** ^1^ Department of Paediatric Surgery, School of Clinical Medicine, Faculty of Health Sciences University of the Witwatersrand Johannesburg South Africa

**Keywords:** anorectal malformations, colorectal, transition of care

## Abstract

**Background:**

Little is known about how patients with anorectal malformations (ARMs), their caregivers and healthcare providers perceive and experience transition from pediatric to adult care (transition of care) in low‐ and middle‐income countries. This study aimed to explore the perceptions and experiences of young adults, adolescents, their caregivers, and healthcare providers regarding transition of care, as well as their perceptions of an ideal transition of care at the Johannesburg Pediatric Colorectal Clinic.

**Methods:**

A qualitative, exploratory study was conducted employing rich pictures. A workshop was held for each of the four stakeholder groups. Participants were asked to draw a rich picture illustrating their perceptions and experiences of the transition of care, followed by a second picture depicting the ideal transition of care. Data were analyzed using Braun and Clarke's thematic analysis.

**Results:**

Four overarching themes were identified: (1) *Born to shine*—living with ARM as a lifelong condition that shapes, but does not define, identity; (2) *Golden gloves*—pediatric services as trusted, emotionally safe spaces, contrasted with fear and uncertainty regarding adult care; (3) *Growing up, letting go*—transition experienced as both developmental progression and relational loss; and (4) *Overwhelmed joint passion*—system fragmentation, limited adult expertise in congenital colorectal conditions, poor information transfer, and reliance on informal pediatric workarounds. Transition of care was experienced as a fragile, relational and system‐level process rather than a discrete transfer event. It was marked by the loss of trusted pediatric relationships, uncertainty regarding adult expertise, and fragmented information transfer. These experiences shaped participants' perceptions of an “ideal transition” as one that is relationally anchored, developmentally appropriate, and coordinated across services. Participants identified feasible, low‐resource strategies, including: adolescent‐focused clinics, joint pediatric adult consultations to build trust, identifiable adult “champions,” and structured information‐handover tools.

**Conclusion:**

Co‐designed transition pathways offer a pragmatic opportunity to strengthen lifelong care for patients with ARMs in resource‐constrained settings.

## Graphical abstract

Little is known about how patients with anorectal malformations (ARMs), their caregivers and healthcare providers perceive and experience transition from pediatric to adult care (transition of care) in low‐ and middle‐income countries. This study aimed to explore the perceptions and experiences of young adults, adolescents, their caregivers, and healthcare providers regarding transition of care, as well as their perceptions of an ideal transition of care at the Johannesburg Paediatric Colorectal Clinic.

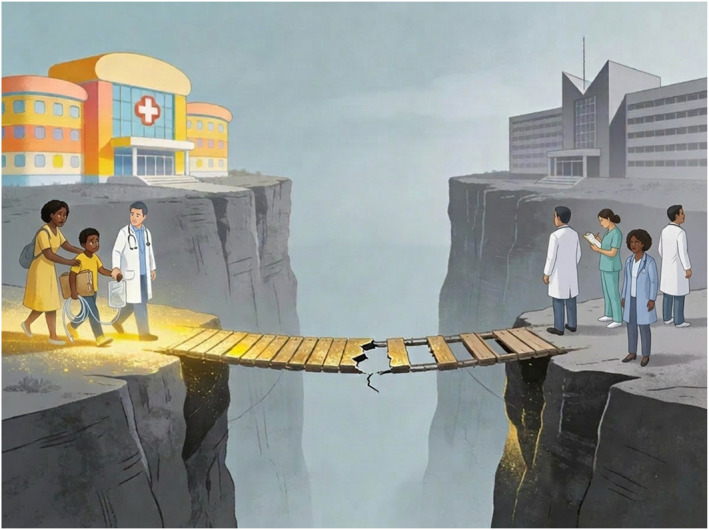

## 1 Introduction

Transition from pediatric to adult healthcare services is a critical juncture for individuals with lifelong, complex health conditions [1]. This transition usually occurs during adolescence, a phase now acknowledged within global health frameworks as a neglected but pivotal window for optimizing health outcomes [2]. Anorectal malformations (ARM) encompass a spectrum of congenital anorectal anomalies that typically require multidisciplinary, longitudinal care spanning childhood into adulthood [3]. Successful transition is associated with improved continuity of care, sustained engagement with services, and outcomes; conversely, poorly managed transition is linked to deterioration in continence and bowel management, missed follow‐up, and an increased risk of adverse health events [4].

In high‐resource settings, structured transition programmes have demonstrated the potential to mitigate care discontinuities [4]. However, transition remains challenging in low‐ and middle‐income countries (LMICs) where health systems often operate in a fragmented manner, with limited integration between pediatric and adult services, workforce shortages, and access barriers [5, 6]. There is a recognized gap in LMICs detailing the experiences of adolescents and young adults with ARM, their caregivers, and healthcare providers during transition [6]. This information is essential to design feasible, patient‐centered transition models suitable for resource‐constrained settings.

The study aimed to explore the perceptions and experiences of young adults, adolescents, their caregivers, and healthcare providers regarding the transition from pediatric to adult care (transition of care), as well as their perceptions of the ideal transition of care.

## 2 Material and Methods

A qualitative, exploratory study was conducted employing rich pictures (a technique from Checkland's Soft Systems Methodology) [7]. This visual and participatory method enables participants to externalize perceptions and experiences that may be difficult to express verbally [8]. It is easier for the intuitive consciousness to communicate with impressions and symbols than with words. Rich pictures or situation summaries are used to depict complicated situations; to reflect the situation in all its richness [9]. The drawing of rich pictures is an “iterative process of understanding and refining of understanding,” opening of dialogue and coming to a broad, shared understanding of a situation [10].

The study was conducted at the Johannesburg Paediatric Colorectal Clinic (JPCC) in the Department of Paediatric Surgery at Chris Hani Baragwanath Academic Hospital in Johannesburg, South Africa. The clinic manages over 400 ARM patients from birth to 35 years of age, as many patients with these congenital conditions who require bowel management have not been able to transition to adult services successfully.

The study sample consisted of adolescents (9–16 years) and young adults (≥ 16 years) with ARM, caregivers of adolescents who attend or have attended the JPCC, and healthcare providers involved in pediatric and adult ARM care. All patient and caregiver participants were currently engaged with care at the JPCC at the time of recruitment. Purposive sampling was used to ensure diverse representation across age, sex, socio‐economic background, and healthcare experiences. Sample adequacy was assessed iteratively during data collection and analysis. After completion of the workshops, the research team reviewed the coding framework and agreed that no substantively new codes were emerging within or across groups, and that the data were sufficient to address the study aim. Patients, caregivers and healthcare providers who were not proficient in English were excluded to allow for real‐time interaction within groups.

Workshops were held for each stakeholder group at a neutral venue in the hospital at convenient times. The workshops were facilitated by clinicians from the pediatric colorectal team at the study site (L.H., N.M.), who were known to many patient and caregiver participants, and by a non‐clinical qualitative researcher (J.S.). We recognize that these dual clinician–researcher roles created power differentials that may have influenced what participants felt comfortable to disclose. To mitigate this, facilitators stressed that participation was voluntary and would not affect clinical care, used open‐ended, non‐directive questions, and invited both positive and critical reflections on services. Written informed consent and assent were obtained from participants as appropriate for participation and the recording of the workshop. Demographic data (patients: age, sex, ARM type, number of surgeries, bowel management; caregivers: age, sex, relationship to patient; providers: age, sex, current employment) were collected. The study and the rich picture methodology were introduced. Participants were then divided into groups of 4–6 participants, and each group was asked to draw a rich picture of their experience of the transition of care. Then each group were asked to explain their rich picture. Following a refreshment break, the groups were asked to draw rich pictures depicting the ideal transition of care and to explain them again. Explanations were audio‐recorded on two mobile phones. Descriptive and reflective field notes were taken during and after the workshops.

The recordings were transferred to a password‐protected laptop immediately after the workshop to ensure secure data storage and confidentiality. One author (N.M.) transcribed the audio recordings verbatim, which were checked by the other authors for accuracy. Rich pictures were scanned and described in field notes, documenting key visual elements, their spatial relationships and any written labels. During analysis, these visual descriptions were examined alongside the corresponding audio‐recordings, integrating what was drawn with what was said. The transcriptions, field notes and rich pictures were analyzed and synthesized using Braun and Clarke's thematic analysis framework [11]. During analysis, the team engaged in discussions about how our clinical commitments might shape coding and theme development, and the non‐clinical team member helped to challenge assumptions.

The study's trustworthiness met the criteria of credibility, dependability, confirmability, transferability and authenticity. Credibility was ensured by the range of participants validating the data by providing their own multiple perspectives of the subject within the group, and by returning the transcripts to participants for final validation. Transferability of the data was enabled by providing context to the environment and population being studied. A detailed description of the methodology allows for dependability. This study ensured authenticity by comprehensively recording data and giving examples of contextual descriptions by participants to illustrate the interpretation of the information collected.

In reporting the results, participants are referred to by the group they were in and a letter within that group.

Reporting of this qualitative study was guided by the Consolidated Criteria for Reporting Qualitative Research (COREQ), and a completed COREQ checklist is provided as Supporting Information S1: Online Resource 1.

## 3 Results

The details of the four workshops are shown in Table 1, and the participants' demographics are shown in Table 2.

**TABLE 1:** Details of the four workshops.

	Workshop 1	Workshop 2	Workshop 3	Workshop 4
	Young adults	Adolescents	Caregivers	Healthcare workers
Number of groups	1	3	2	1
Number of participants per group	6	4, 5, 5	6, 7	6
Duration of workshops hour:minutes	1:45	2:45	2:45	2:30

**TABLE 2:** Demographics of participants.

Variable	Caregivers, *n* = 13	Healthcare workers, *n* = 6	
	*Mean* (*SD*)	*Mean* (*SD*)	
Age	44 (10)	42 (8)	
	*n (%)*	*n (%)*	
Sex			
Female	6 (100)	6 (100)	
Relationship to patient			
Mother	12 (92)		
Grandmother	1 (8)		
Current employment		Pediatric colorectal surgeon	1 (16.7)
		Pediatric colorectal fellow	1 (16.7)
		Adult colorectal surgeon	1 (16.7)
		Gynecologist	1 (16.7)
		Enrolled nurse	1 (16.7)
		Professional nurse	1 (16.7)

Variable	Young adults, *n* = 6		Adolescents, *n* = 14	
	*Mean (SD)*		*Mean (SD)*	
Age	23 (6)		13 (2)	
	*n (%)*		*n (%)*	
Sex				
Female	2 (33%)		9 (64%)	
ARM type	Recto‐bladder neck	1 (16.7)	Rectovestibular	5 (35.7)
	Unknown	5 (83.3)	Cloaca	2 (14.3)
			Rectoperineal	2 (14.3)
			No fistula	2 (14.3)
			Unknown	2 (14.3)
			Cloacal exstrophy	1 (7.1)
	*Mean (SD)*		*Mean (SD)*	
Number of surgeries	7 (3)		6 (4)	
Bowel management, *n* (%)	ACE washouts	5 (83.3)	None	5 (35.7)
	None	1 (16.7)	Laxatives	3 (21.4)
			Retrograde washouts	3 (21.4)
			ACE washouts	3 (21.4)

**FIGURE 1:**
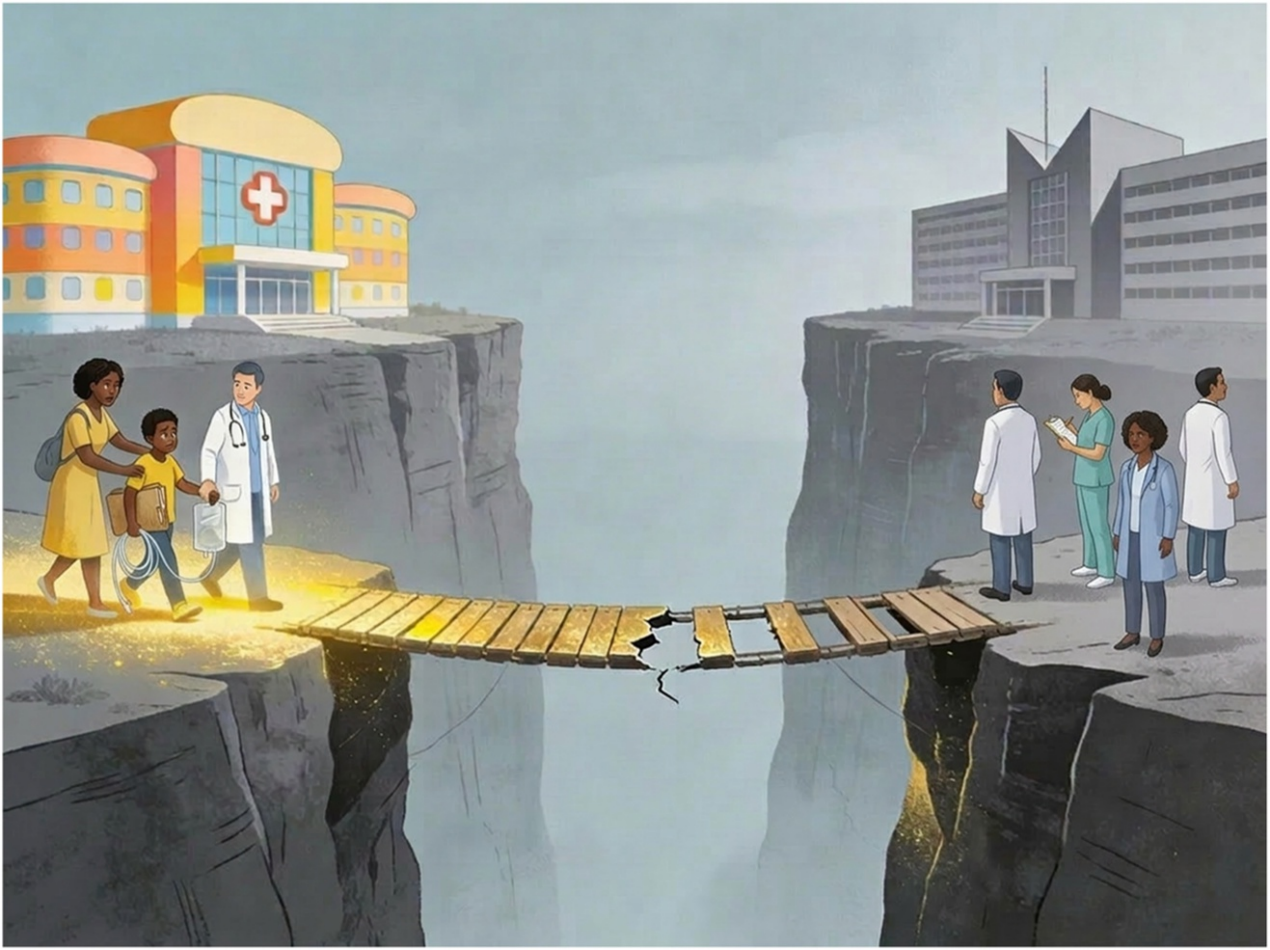
Transition of care: crossing a fragile bridge.

The synthesized data revealed a metaphor (Figure 1) depicting the transition of care as crossing a fragile bridge rather than stepping onto a well‐built road, highlighting the movement from pediatric to adult services as risky, uncertain, and dependent on pediatric champions. The anxious adolescent, carrying his folder and washout set, nudged forward by his mother and guided by the pediatric surgeon, contrasted with fragmented, individually focused adult clinicians on the far side, underscoring emotional, relational, and systemic gaps that make this crossing precarious.

We identified four themes that map how participants made sense of this journey: “Born to shine”—living with an ARM and growing up; “Golden gloves”—safe spaces and fragile trust; “Growing up, letting go”—transition as loss and possibility; and “Overwhelmed joint passion”—system strain, workarounds, and efforts to build a more reliable bridge into adult care.

### 3.1 Theme 1: “Born to Shine”—Living With ARM and Growing Up

Participants described ARM as a lifelong condition that shaped—but did not fully define—their identities. Patients emphasized resilience, fluctuating emotions, and aspirations, positioning ARM within broader developmental processes. In the adolescent male group's “Christiano Ronaldo” picture (Supporting Information S1: Online Resource 2), suns and clouds presented transient challenges. One participant stated, “Sometimes I feel lonely… sometimes angry… sometimes sad… sometimes I feel strong, knowing I'll still be okay at the end of the day,” (A1.1P1) while another noted, “The clouds are the problems that come and go, but the sun is forever shining” (A1.1P5).

Adolescent girls' pictures, including “The Clean Girls” and “The Happiness of Girls” (Supporting Information S1: Online Resources 3 and 4), used bright colors, stars, sweets, fruit, hearts, and paired figures to portray kindness, cohesion, and joy, alongside hidden vulnerability. They described themselves as “sweet,” “magical,” (A2.1P2) and mutually supportive, but recognized that distress was often concealed: “we just thought that we must show our happiness” (A3.1P1).

Across groups, independent bowel management was tightly linked to future possibilities. In “The Future Lies in Our Hands” (Supporting Information S1: Online Resource 7), adolescents drew large hands, a brain representing learning, cars to illustrate responsibility and forward progress. One adolescent stated, “You must take responsibility of your future so that you cannot beg anyone,” (A2.2P2) linking self‐management to education and work.

Caregivers similarly positioned their children as “future‐driven,” surrounding images of homes and children with hearts, suns, and stars around, and articulating aspirations such as becoming doctors, dancers, or athletes. One caregiver reflected, “This here is my two children. And this here is my heart. My life, my pains, my laughs, my struggles and everything I'm going through is in this heart with my two children,” (C1.1P5), underscoring how child well‐being underpinned caregiver identity.

Young adults described a shift from childhood shame and isolation to greater autonomy and social participation. In the “Our Journey of Life” (Supporting Information S1: Online Resource 8) long roads, hospitals, and a prominent ACE syringe depicted years of travel, delayed access to care, and the centrality of bowel management. Participants recalled bullying: “when I was being bullied I was way younger” (YA1.2P2) and contrasted this with the impact of effective management: “After the ACE I'm happy. I can do all my activities without worrying if I'm going to mess on myself” (YA1.1P2). These narratives suggest that transition is not only a change of clinical provider, but also a psychologically significant phase in which young people renegotiate identity, moving from childhood experiences of bullying and shame toward integrating life with an ARM into an emerging adult self.

Caregivers reinforced this developmental trajectory; one reported that her daughter had avoided leaving home for years because of scars, but after surgery, “you can't even find her in the house, she is outside playing” (C1.1P6).

Overall, patients presented themselves as resilient individuals “born to shine,” using bowel management as a gateway to participation and imagined adult futures.

### 3.2 Theme 2: “Golden Gloves”—Safe Spaces and Fragile Trust

Pediatric services were consistently described as safe spaces characterized by familiarity and trust. In contrast, adult services were anticipated as unfamiliar and fragmented. Young adults described needing to repeatedly explain their condition to adult providers and accept a shift from personalized support to task‐focused interactions.

Adolescents' drawings contained hands, hearts, and enclosed spaces symbolizing protection and care. One adolescent explained, “we are saying that the team cares about our futures” (A2.1P3), and another described pediatrics as a place they “feel safe” (A3.2P4).

Caregivers emphasized long‐standing, personalized relationships in pediatrics. One recounted, “I only got help here at Bara,” (C1.1P7) while another reflected, “And now here, he is being handled with golden gloves, silver spoon. Now on the other side it won't be like that. And definitely he is going to deteriorate. And he will think this is a different hospital, and these people are not working the same so there is a likelihood he can be depressed. Not knowing that he is the one at fault” (C2.1P8). These narratives linked continuity and familiarity with clinical and psychological stability.

Anticipation of adult care generated anxiety about rebuilding trust. One participant remarked, “I would like the doctors to treat us the same way they treated us at the children's clinic. Because they were much more kinder. And in future we don't know how they will be” (A3.2P1). Caregivers added that adult providers might lack both technical expertise in congenital conditions and the relational sensitivity of pediatric providers.

Healthcare workers described their own difficulty relinquishing patients: “But in reality we can't let them go because the adults don't know how to manage them. So we've had 20‐ and 22‐year‐olds in our clinic because we can't let them go” (H1.1P3).

Participants proposed joint consultations and gradual introductions to adult teams as strategies to preserve trust, but these remained aspirational rather than standard practice.

### 3.3 Theme 3: “Growing Up, Letting Go”—Transition as Loss and Possibility

Transition was experienced as an extended process rather than a single event. Adolescents focused on the loss of “kind and sweet” clinicians and called for developmentally tailored services. One said, “It's really sad … I feel upset because they are changing the doctors,” and added, “we want doctors to create a new design like a new clinic … because we want to be treated like our age” (A2.1P2). These preferences were visualized in “The Future Lies in Our Hands” (Supporting Information S1: Online Resource 7), they drew a separate teenage building and a familiar nurse. Young adults echoed this anticipated rupture: “If they said from tomorrow you have to go to adults? Yoh, sad, very sad” (YA1.2P1).

Caregivers framed transition as “letting go” of children intensively supported in pediatrics, worrying about advocacy and understanding their children once they themselves were absent. One asked: “We are planning for the future for our kids…my worries are when I die, who is going to take care of them? Who is going to understand what they are going through?” (C1.1P1). For parents of girls, future relationships and reproduction were prominent concerns: “What will happen when she starts dating? Will she ever be able to find love … to give birth naturally … will her kids have the same problem?” (C1.1P2).

Healthcare workers highlighted structural discontinuities. One explained, “When they are little we take care of them … when it comes here there is a gap … when they are old they are standing alone, no one is handing over to anyone,” (H1.1P5) and drew a sequence of figures with a break in the line which colleagues interpreted as “no transition of care.” Caregivers advocated to remain involved during early adulthood: “Please give us the opportunity to escort them at least until the age of 18 … at 16 they are still blind” (C1.2P2). Across accounts, transition emerged as a contested process of “growing up and letting go,” balancing autonomy, protection, and system gaps.

### 3.4 Theme 4: “Overwhelmed Joint Passion”—System Realities and Workarounds

Healthcare workers' located transition within broader resource and organizational constraints. In “Overwhelmed Joint Passion” (Supporting Information S1: Online Resource 13) they drew a bulging file, an abdomen covered in scars and arrows pointing to multiple organ systems, alongside a globe indicating international searches for advice. This mirrored their description of “difficult patients, difficult histories, difficult files, difficult handwriting, difficult surgical history … multiple problems that make them cry because they don't know how to face that because it's gynae, it's colorectal, it's GIT, it's urology, it's pregnancy, it's fertility, it's a lot of things that overwhelm them” (H1.1P1). They stressed that this work extended beyond contracted time, describing “spending money on them, your own personal money, your own personal time” (H1.1P1).

Broken lines and disconnected hospitals represented weak information systems and fragmented pathways. Clinicians noted that patients moving between private and state sectors often arrived without usable records: “But then you ask them, and they have nothing. Absolutely nothing. Like no information. Most of them don't even know the type of malformation, they don't even know, like nothing” (H1.1P1). Adult surgeons in the state sector were depicted at the margins: “We've tried very hard to get the adult surgeons in the state sector to come. We beg and we plead and I know for us this is our whole world, and for the adult surgeons, it's a tiny little part of their world” (H1.1P3).

In contrast, the “ToCollaboration” (Supporting Information S1: Online Resource 14) captured a desired shift from individual heroics to structured, multidisciplinary care. Healthcare workers drew a large MDT table, a calendar for a dedicated transition clinic day, and icons for electronic records, with pediatric and adult clinicians side by side. One summarized: “So I think we need champions. I don't know how we get that. Maybe you need to develop relationships with people individually, see if it's their interest. I'm not sure. I do think we need all the departments together to work for these patients for them to do well” (H1.2P4). They proposed identifying adult “champions,” establishing a scheduled transition clinic, and using simple electronic or patient‐held summaries at discharge to create a more coherent pathway, emphasizing that coordination and commitment, rather than sophisticated technology, were the key requirements.

## 4 Discussion

Across all groups, transition was perceived and experienced as a relational, developmental and system‐level process that is currently fragile and poorly supported. Patients described moving from childhood isolation, bullying and shame toward greater freedom and self‐advocacy after bowel management interventions, yet feared losing the safe spaces and trusted relationships developed with pediatric teams. Caregivers expressed parallel hopes and anxieties about education, work, fertility, and independence, while healthcare workers felt overwhelmed by complex, resource‐intensive patients in a fragmented system, yet remained deeply committed to them. In imagining an ideal transition, participants suggested that transition pathways for ARM must deliberately integrate developmental timing, psychosocial support and system design, rather than focusing solely on age‐based transfer.

Participants framed a good transition as preserving relational continuity and specialist knowledge across the life‐course, consistent with consensus recommendations that transition for ARM should be planned, multidisciplinary, and initiated early, with clear allocation of responsibility [1, 4, 12]. Central to this was trust, built over years of longitudinal pediatric care. The accounts also highlight implicit power dynamics between pediatric and adult systems: pediatric teams, who “know” these patients, often function as gatekeepers and advocates, deciding when and whether to relinquish care to adult services perceived as less prepared or less invested. This asymmetry reinforces the dependence of transition on a small number of pediatric champions.

Prior studies have highlighted limited structured transition, inconsistent long‐term follow‐up and the lack of formal adult‐service responsibilities, despite ARM being lifelong [1, 4, 13, 14]. The strong preference for continued pediatric follow‐up, even into the third decade of life, appeared a rational response to where trust and expertise were perceived to reside, and echoes international findings that many adults with ARM remain tied to pediatric teams or are lost to follow‐up rather than integrated into adult services [15, 16, 17]. These findings suggest that trust is not automatically transferable at handover and that access to the pediatric colorectal team at key life events (e.g., pregnancy or major surgery) may be more important than a rigid age cut‐off.

Adolescents clearly wished to be seen as “bigger children” in age‐appropriate spaces while retaining the kindness and safety of pediatrics, aligning with literature emphasizing respect and feeling “taken seriously” during transition [18]. Existing work on ARM and Hirschsprung disease underscores the need to build self‐management skills and gradually shift responsibility from parents to young people [15, 19]. In contrast, caregivers in this study often felt their children remained “blind” at 16 and requested accompaniment to 18, reflecting concerns about non‐disclosure of symptoms, stigma and risky decision‐making; similar ambivalence about “letting go” is described in high‐income settings [1, 12]. Fertility, sexual function and future relationships were particularly salient and were viewed as essential to ideal follow‐up. Outcome document impaired sexual function, altered body image and variable fertility among adults with ARM, and highlight the need for gynecological and urological input, yet surveys suggest these topics are inconsistently addressed [17, 20, 21]. Our findings support calls to embed structured, age‐appropriate discussions of sexuality and reproduction as a routine component of ARM transition clinics, rather than ad hoc crisis‐driven discussions [1, 12].

Healthcare workers vividly described system‐level obstacles, including illegible or absent notes, lack of integrated records across state and private sectors, limited participation of adult surgeons in transition activities, and constrained access to investigations and fertility interventions. Similar documentation gaps, poor information continuity, and weak pediatric–adult linkages have been described in other LMIC surgical and chronic disease contexts, where paper‐based records predominate and patients move between sectors. Access to specialized colorectal care is further shaped by broader structural inequities in LMIC health systems. In this context, adolescents' and caregivers' fears about “falling through the cracks” are not only relational but also grounded in material constraints that disproportionately affect marginalized patients.

Participants proposed primarily organizational solutions: a designated transition clinic day with adult and pediatric clinicians present or readily available; simple electronic or patient‐held discharge summaries; better use of existing multidisciplinary meetings; and a small group of adult “champions” for congenital colorectal conditions. Comparable models, including joint clinics, structured transition meetings, and patient‐held records, have been successfully implemented in other chronic pediatric conditions without major new infrastructure [22, 23, 24]. Linking these efforts to local audit and research could help embed ARM‐specific transition knowledge within adult surgical training and support context‐appropriate guidance.

A key strength of this study is its inclusion of adolescents, young adults, caregivers and healthcare workers in jointly describing both current transition experiences and an ideal model of transition, with participatory visual methods generating rich data.

This study was conducted in a single tertiary center with a specialized pediatric colorectal clinic, which has implications for the transferability of the findings to other LMIC settings and to non‐specialized institutions. The JPCC is characterized by a relatively stable multidisciplinary team, a high volume of ARM patients, and longstanding relationships between clinicians and families, features that may support the development of informal “workarounds” and pediatric champions. In more decentralized or resource‐constrained settings without dedicated colorectal services, transition pathways may be even more fragmented, and the relational and informational gaps we describe may be amplified. At the same time, the organizational solutions proposed by participants are low‐cost and could be adapted within a range of health system configurations.

A further limitation is that all patient and caregiver participants were actively engaged in follow‐up at the JPCC. Young people who have disengaged from care, or who never reach specialist services, may experience transition differently—for example, encountering more abrupt transfers, greater diagnostic uncertainty, or longer periods without bowel management support. Future work is needed to purposively include adolescents and adults with ARM who are lost to follow‐up or managed outside specialist centers to better understand these trajectories.

Our exclusion of non‐English speakers at a multilingual public hospital is another important limitation. The decision was pragmatic, but it likely skewed the sample toward participants with higher levels of formal education and English proficiency. Experiences of transition among non‐English speaking families, who may face additional communication barriers are therefore under‐represented in our data.

## 5 Conclusion

Participants experienced transition not as a discrete transfer, but as a relational, developmental and system‐level process that is currently fragile, fragmented and reliant on a few pediatric champions. This multi‐stakeholder study from a resource‐constrained setting shows how gaps in adult expertise, documentation, communication, and practical support undermine global aspirations for structured, lifelong ARM follow‐up and shift the emotional and organizational burden onto pediatric teams. At the same time, participants proposed feasible, ARM‐specific design elements—adolescent‐focused joint pediatric–adult clinics, identifiable adult “champions,” and simple handover tools—that align with international guidance and could realistically strengthen lifelong ARM care in South Africa and similar resource‐constrained settings.

## Author Contributions

**Leila Hartford:** conceptualization, writing – original draft, formal analysis, writing – review and editing, visualization. **Niveshni Maistry:** data curation, project administration. **Giulia Brisighelli:** conceptualization, writing – review and editing. **Juan Scribante:** conceptualization, methodology, writing – review and editing, supervision.

## Funding

The authors have nothing to report.

## Ethics Statement

This study was approved by the University of the Witwatersrand Human Research Ethics Committee (M250626).

## Consent

Informed consent and assent were obtained from all individual participants included in the study.

## Conflicts of Interest

The authors declare no conflicts of interest.

## Supporting information


Supporting Information S1

## Data Availability

The data that support the findings of this study are available from the corresponding author upon reasonable request.
