# Supplementary material for: Transition of Care From Pediatric to Adult Services for Patients With Anorectal Malformations: A Qualitative Study
Source: World J Surg. 2026 Jun 25;50(8):2372–9. doi: 10.1002/wjs.70467 (PMC13460811; doi:10.1002/wjs.70467)
Supplement: Supplementary file 1 [file WJS-50-2372-s001.docx]

**Supplementary material**

**Online Resource 1 (COREQ Checklist)**

**Domain 1: Research team and reflexivity**

**Personal characteristics**

| **Item** | **Guide question / description** | **Location in revised manuscript** | **Response** |
| --- | --- | --- | --- |
| 1 | Interviewer/facilitator. Which author/s conducted the interview or focus group? | Methods | Workshops were facilitated by LH, NM, and JS |
| 2 | Credentials. What were the researchers’ credentials? | Title page | LH: MBBCh, MSc, MMED, FCPS(SA); NM: MBBCh, MMED, FCPS(SA); GB: MD, FCPS(SA); JS: PhD |
| 3 | Occupation. What was their occupation at the time of the study? | Methods / title page | LH and NM were clinicians from the paediatric colorectal team; JS was a non-clinical qualitative researcher |
| 4 | Gender. Was the researcher male or female? | Not stated | Not explicitly stated for all authors, although participant sex is reported in Table 2. |
| 5 | Experience and training. What experience or training did the researcher have? | Author contributions / Methods | The team included clinicians experienced in paediatric colorectal care and a researcher with methodological and supervisory expertise in qualitative research. |

**Relationship with participants**

| **Item** | **Guide question / description** | **Location in revised manuscript** | **Response** |
| --- | --- | --- | --- |
| 6 | Relationship established. Was a relationship established prior to study commencement? | Methods | Yes. Facilitators from the paediatric colorectal team were known to many patient and caregiver participants |
| 7 | Participant knowledge of the interviewer. What did the participants know about the researcher? | Methods | Participants would have known some facilitators as clinicians at the study site; the manuscript explicitly acknowledges the dual clinician–researcher roles. |
| 8 | Interviewer characteristics. What characteristics were reported about the interviewer/facilitator? | Methods | The manuscript includes a reflexivity statement describing the facilitators’ clinical roles, power differentials, and mitigation strategies. |

**Domain 2: Study design**

**Theoretical framework**

| **Item** | **Guide question / description** | **Location in revised manuscript** | **Response** |
| --- | --- | --- | --- |
| 9 | Methodological orientation and theory. What methodological orientation underpinned the study? | Methods | Qualitative exploratory study using rich pictures from Checkland’s Soft Systems Methodology and Braun and Clarke’s thematic analysis. |

**Participant selection**

| **Item** | **Guide question / description** | **Location in revised manuscript** | **Response** |
| --- | --- | --- | --- |
| 10 | Sampling. How were participants selected? | Methods | Purposive sampling was used to ensure diversity across age, gender, socio-economic background, and healthcare experiences. |
| 11 | Method of approach. How were participants approached? | Methods | Participants were recruited from the JPCC and invited to workshops; the exact method of approach is not described in detail. |
| 12 | Sample size. How many participants were in the study? | Results, Tables 1 and 2 | Total n=39: young adults n=6, adolescents n=14, caregivers n=13, healthcare workers n=6. |
| 13 | Non-participation. How many people refused to participate or dropped out? | Not stated | Not reported in the revised manuscript. |

**Setting**

| **Item** | **Guide question / description** | **Location in revised manuscript** | **Response** |
| --- | --- | --- | --- |
| 14 | Setting of data collection. Where was the data collected? | Methods | Workshops were held for each stakeholder group at a neutral venue in the hospital. |
| 15 | Presence of non-participants. Was anyone else present besides participants and researchers? | Not stated | Not reported in the revised manuscript. |
| 16 | Description of sample. What are the important characteristics of the sample? | Results, Table 2 | Demographic data included age, sex, ARM type, number of surgeries, bowel management, caregiver relationship to patient, and current employment of healthcare workers. |

**Data collection**

| **Item** | **Guide question / description** | **Location in revised manuscript** | **Response** |
| --- | --- | --- | --- |
| 17 | Interview guide. Were questions, prompts, guides provided? Was it pilot tested? | Methods | Participants were asked to draw a rich picture of current transition experiences and a second of the ideal transition, then explain each; pilot testing is not reported. |
| 18 | Repeat interviews. Were repeat interviews carried out? | Methods | No repeat interviews were reported. |
| 19 | Audio/visual recording. Did the research use audio or visual recording? | Methods | Yes. Explanations were audio-recorded, and rich pictures were used as visual data. |
| 20 | Field notes. Were field notes made? | Methods | Yes. Descriptive and reflective field notes were taken during and after the workshops. |
| 21 | Duration. What was the duration of the interviews or focus group? | Results, Table 1 | Workshop duration ranged from 1 hour 45 minutes to 2 hours 45 minutes. |
| 22 | Data saturation. Was data saturation discussed? | Methods | Yes. Sample adequacy was assessed iteratively, and the team agreed that no substantively new codes were emerging within or across groups. |
| 23 | Transcripts returned. Were transcripts returned to participants for comment and/or correction? | Methods | Yes. The manuscript states that credibility was supported by returning transcripts to participants for final validation. |

**Domain 3: Analysis and findings**

**Data analysis**

| **Item** | **Guide question / description** | **Location in revised manuscript** | **Response** |
| --- | --- | --- | --- |
| 24 | Number of data coders. How many data coders coded the data? | Methods | The exact number of coders is not explicitly stated, although the manuscript indicates team-based analysis and author review of transcripts. |
| 25 | Description of the coding tree. Did authors provide a description of the coding tree? | Results / Methods | A formal coding tree is not presented, but four overarching themes are described and linked to the analysis. |
| 26 | Derivation of themes. Were themes identified in advance or derived from the data? | Methods | Themes were derived from the data using Braun and Clarke’s thematic analysis framework. |
| 27 | Software. What software, if applicable, was used to manage the data? | Not stated | No qualitative data analysis software is reported. |
| 28 | Participant checking. Did participants provide feedback on the findings? | Methods | Partial participant checking is reported through transcript validation; participant feedback on developed themes is not specifically described. |

**Reporting**

| **Item** | **Guide question / description** | **Location in revised manuscript** | **Response** |
| --- | --- | --- | --- |
| 29 | Quotations presented. Were participant quotations presented to illustrate the themes/findings? Was each quotation identified? | Results | Yes. Quotations are presented and identified by stakeholder group and participant code. |
| 30 | Data and findings consistent. Was there consistency between the data presented and the findings? | Results / Discussion | Yes. Findings are illustrated with quotations, visual data, and linked interpretively to the four themes. |
| 31 | Clarity of major themes. Were major themes clearly presented in the findings? | Results | Yes. Four overarching themes are clearly named and described. |
| 32 | Clarity of minor themes. Is there a description of diverse cases or discussion of minor themes? | Results / Discussion | Yes, to a moderate extent. Variation across stakeholder groups and contrasting accounts are included, although minor themes are not separately tabulated. |

**Online resources 2 –14 (Rich Pictures)**

**Key to verbatim quote coding:**

Stakeholder: Adolescent [A], Young adults [YA], Caregiver [C], Healthcare worker [H]

Participant group: 1-4

Session: Current transition [1], ideal transition [2]

Participant number: 1-7

E.g.: A1.2.4 = Adolescent group 1, session 2, participant 4

**Adolescents Session 1 (Current Transition Experience):**

Online Resource 2: A1.1 Christiano Ronaldo

Online Resource 3: A2.1 The Clean Girls

Online Resource 4: A3.1 The Happiness of Girls

**Adolescents Session 2 (Ideal Transition):**

Online Resource 5: A1.2 How Doctors Can Help Us

Online Resource 6: A2.2 Doctors Love Us

Online Resource 7: A3.2 The Future Lies in Our Hands

**Young Adults Session 1 (Current Transition Experience):**

Online Resource 8: YA1.1 Our Journey of Life

**Caregivers Session 1 (Current Transition Experience):**

Online Resource 9: C1.1 Everyone has a Role to Play

Online Resource 10: C2.1 Future Plans

**Caregivers Session 2 (Ideal Transition):**

Online Resource 11: C1.2 Ideas

Online Resource 12: C2.2 Opportunities

**Healthcare workers Session 1 (Current Transition Experience):**

Online Resource 13: H1.1 Overwhelmed Joint Passion

**Healthcare workers Session 2 (Ideal Transition):**

Online Resource 14: ToCollaboration
